# Long-term single-lead electrocardiogram monitoring to detect new-onset postoperative atrial fibrillation in patients after cardiac surgery

**DOI:** 10.3389/fcvm.2022.1001883

**Published:** 2022-09-23

**Authors:** Kang He, Weitao Liang, Sen Liu, Longrong Bian, Yi Xu, Cong Luo, Yifan Li, Honghua Yue, Cuiwei Yang, Zhong Wu

**Affiliations:** ^1^Department of Cardiovascular Surgery, West China Hospital of Sichuan University, Chengdu, China; ^2^Center for Biomedical Engineering, School of Information Science and Technology, Fudan University, Shanghai, China

**Keywords:** postoperative new-onset atrial fibrillation, long-term ECG monitoring, P wave characteristics, machine learning, support vector machine

## Abstract

**Background:**

Postoperative atrial fibrillation (POAF) is often associated with serious complications. In this study, we collected long-term single-lead electrocardiograms (ECGs) of patients with preoperative sinus rhythm to build statistical models and machine learning models to predict POAF.

**Methods:**

All patients with preoperative sinus rhythm who underwent cardiac surgery were enrolled and we collected long-term ECG data 24 h before surgery and 7 days after surgery by single-lead ECG. The patients were divided into a POAF group a no-POAF group. A clinical model and a clinical + ECG model were constructed. The ECG parameters were designed and support vector machine (SVM) was selected to build a machine learning model and evaluate its prediction efficiency.

**Results:**

A total of 100 patients were included. The detection rate of POAF in long-term ECG monitoring was 31% and that in conventional monitoring was 19%. We calculated 7 P-wave parameters, Pmax (167 ± 31 ms vs. 184 ± 37 ms, *P* = 0.018), Pstd (15 ± 7 vs. 19 ± 11, *P* = 0.031), and PWd (62 ± 28 ms vs. 80 ± 35 ms, *P* = 0.008) were significantly different. The AUC of the clinical model (sex, age, LA diameter, GFR, mechanical ventilation time) was 0.86. Clinical + ECG model (sex, age, LA diameter, GFR, mechanical ventilation time, Pmax, Pstd, PWd), AUC was 0.89. In the machine learning model, the accuracy (Ac) of the train set and test set was above 80 and 60%, respectively.

**Conclusion:**

Long-term ECG monitoring could significantly improve the detection rate of POAF. The clinical + ECG model and the machine learning model based on P-wave parameters can predict POAF.

## Introduction

Postoperative atrial fibrillation (POAF) after cardiac surgery was defined as a new episode of atrial fibrillation or flutter requiring treatment after surgery. A more specific definition is a new episode of atrial fibrillation with duration ≥ 5 min detected on any monitoring instrument, including rhythm monitor/telemetry/electrocardiogram, and requiring treatment. POAF is the most common complication after cardiac surgery and is a challenge for cardiac surgeons (1). Patients with POAF are often associated with serious early complications, including hemodynamic instability, acute heart failure and stroke. The incidence of long-term atrial fibrillation and stroke risk is 2 or 3 times that of patients without POAF, accompanied by a significantly increased incidence of heart failure, rehospitalization rate, etc.

Studies have shown that there may be minimal fibrillation, despite a normal sinus rhythm on ECG, which cannot reflect atrial function (2, 3). The electrophysiology and structural reconstruction of the heart occurs before the appearance of the characteristic ECG waveform change in patients prone to AF (4, 5). However, it’s difficult for humans to recognize non-sinus electrical activity, which has become the basis for the prediction of AF.

The rapid development of mobile medical techniques and wearable ECG technology provides a cheap choice for AF detection and assessment. Convenient intermittent or systematic monitoring of elderly patients with specific risk factors can improve the detection of AF (6). The advent of the era of big data makes artificial intelligence applications in medicine possible. Due to its powerful predictive potential, machine learning and deep learning can identify individuals likely to develop AF from their sinus rhythm records (7), which can make clinical practice more effective, convenient and personalized for the identification and prediction of atrial fibrillation.

In conclusion, the purpose of this study was to build a statistical model and machine learning model to predict POAF in patients with preoperative sinus rhythm after cardiac surgery using portable long-term ECG monitoring.

## Patients and methods

This study was approved by the West China Hospital of Sichuan University ethics committee. This study adheres to the Strengthening the Report of Observation Studies in Epidemiology (STROBE) guidelines.

### Patients

Patients with preoperative sinus rhythm undergoing cardiac surgery from September 2020 to December 2021 in the Department of Cardiovascular, West China Hospital of Sichuan University were included. Eligible patients underwent ECG recording 24 h or more before surgery and 7 days or more after surgery by long-term single-lead electrocardiogram. Patients were excluded if they underwent emergency surgery or had paroxysmal atrial fibrillation.

### Electrocardiogram monitoring

The main equipment used in this study is the long-term single-lead electrocardiogram. It is a patch with 2 electrodes 10 cm apart placed over the patients’ precordium at an angle of 45^°^. The device can continuously record and collect ECG data dynamically for up to 14 days.

### Electrocardiograms

**FIGURE 1:**
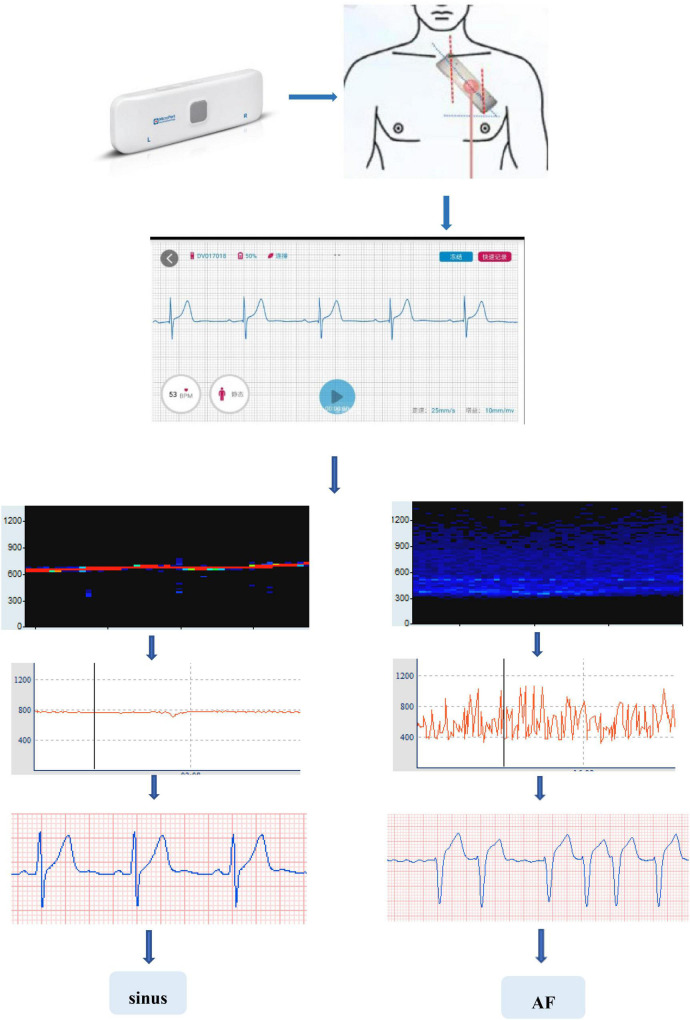
Work flow of portable long-time single-lead ECG monitor and Ecglab.

All raw data were processed by Ecglab software developed by Shanghai Yuanxin Medical (Figure 1). Blinded to the patient outcomes, two readers (KH and SL) labeled and measured the required ECG fragments.

**FIGURE 2:**
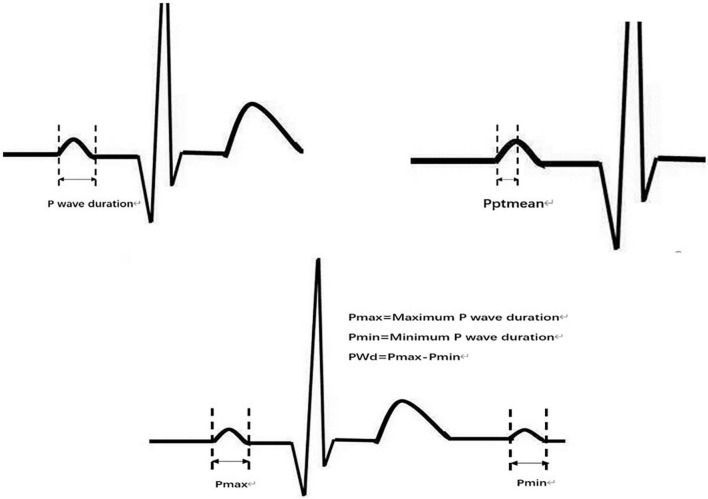
Part of P-wave parameters. P wave duration, Pmax, Pmin, Pptmean, PWd.

The original data were low-pass filtered by Ecglab software and then high-pass filtered to remove noise and correct filter delay. The second step was to smooth the ECG curve and facilitate the algorithm selection. Then, P wave localization (including the start point, first peak point, and end point) was performed based on the ECG waveform localization algorithm provided in a previous study (8), and the positioning results were manually verified. The measurement of the start point was taken at the intersection of the start point of the P wave and the iso-potential line, and the measurement of the end point was taken at the intersection of the end point of the P wave and the iso-potential line. Based on the results of P-wave localization and combined with the reports in the literature (8), 7 P-wave characteristics were calculated: Pmax, Pmin, Pmean, Pstd, P wave dispersion (PWd), Pptmean, and Pptstd. Pmax was defined as the widest P-wave in the selected segment. Pmin was defined as the minimum p-wave time in the fragment. Pmean was defined as the mean p-wave time of all fragments. Pstd was defined as the standard deviation (SD) of P-wave time. PWd was defined as the difference between the maximum and minimum P-wave times. Pptmean was defined as the mean time interval from the start point of the P wave to the peak point. Pptstd was defined as the SD of the p-wave peak time (Figure 2).

## Statistical analysis and machine learning

### Statistical analysis

All statistical analyses were performed using SPSS 25.0 software. All continuous data with a normal distribution are expressed as the mean **±** *SD*, which were assessed by two independent sample *t*-tests. Non-normally distributed continuous data were represented by the median and interquartile range (IQR) and compared with the Mann–Whitney *U*-test. Categorical data are reported as an absolute number and percentage and compared using the chi-square test or Fisher’s exact test. The association between predictors and POAF was assessed by univariate logistic regression separately and combined by multivariate logistic regression. Clinical data and ECG parameters were used to construct prediction models, which were evaluated by receiver operating characteristic curves (ROCs) and areas under the curve (AUCs). All results were two-tailed statistically significant with *P* < 0.05, and confidence intervals (CIs) were calculated at the 95% level.

### Machine learning

Seven P-wave features were calculated, and a few patients’ ECGs that were difficult to recognize by computer were excluded. A total of 94 patients’ data feature matrices (94 × 7) were incorporated into the machine learning model. There were 28 cases in POAF group (Class 1) and 66 cases in no-POAF group (Class 0).

19 cases in Class 1 were randomly selected as the training set, and 9 cases were selected as the test set at a ratio of 7:3. To reduce the overlearning of one group, 19 patients in Class 0 were also randomly selected as the training set, and the remaining 47 patients were selected as the test set. Two schemes were adopted: Scheme A: all patient data were used, training set: 19 cases (0) + 19 cases (1); test set: 47 cases (0) + 9 cases (1); Scheme B: partial patient data were randomly used to ensure data balance as far as possible, training set: 19 cases (0) + 19 cases (1); test set: 9 cases (0) + 9 cases (1).

Support vector machine (SVM) was selected in this experiment. Based on the training set, the optimal hyperparameters of the model (C-penalty factor, gamma-kernel function) were determined by fivefold cross-validation and grid search. The confusion matrix was used to calculate the accuracy (Ac), sensitivity (Sen), and specificity (Spe) of the training set and test set.

## Results

**FIGURE 3:**
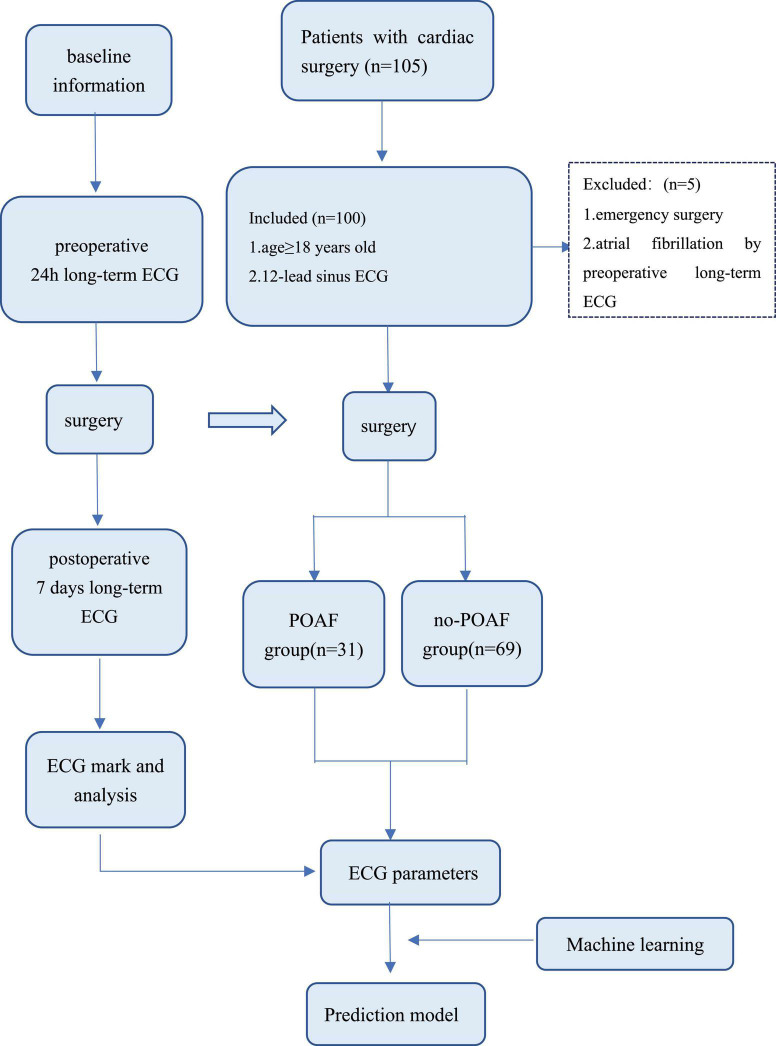
Study flowchart. ECG, electrocardiogram; POAF, postoperative atrial fibrillation.

The preoperative ECG of 105 patients was monitored; however, 5 patients were excluded because paroxysmal atrial fibrillation was detected by preoperative long-term monitoring. A total of 100 patients entered this study (Figure 3). After processing the long-term ECG of 100 patients, we found that POAF occurred in 31 patients, with a detection rate of 31%. Among the 31 patients with POAF, 12 were not identified in the intensive care unit (ICU) or ward, and the detection rate of POAF in routine care was 19%.

The patients’ demographics and information about the operative procedures are shown in Table 1. Of the 100 patients included, 51.6 and 62.3% were males in the POAF and no-POAF groups, respectively. The mean age was 55.97 ± 8.49 years and 50.43 ± 10.59 years in the two groups. Preoperative transthoracic echocardiography (TTE) showed that the left atrium diameter (LAD) in the POAF group was significantly larger than that in the no-POAF group [55.97 ± 8.49 y vs. 50.43 ± 10.59 y, 95% CI (1.25, 9.82), *P* = 0.012]. In addition, the glomerular filtration rate (GFR) (82.02 ± 14.94 ml/min/1.73 m2 vs. 93.00 ± 13.81 ml/min/1.73 m2, *P* < 0.001) and blood urea level (6.40 ± 1.75 mmol/L vs. 5.59 ± 1.32 mmol/L, *P* = 0.012) were significantly different.

**TABLE 1:** Patient demographic, clinicopathologic, and operative characteristics.

Variable	no-POAF (*n* = 69)	POAF (*n* = 31)	*P*-value
Gender, male (%)	43 (62.3%)	16 (51.6%)	0.314
Age (y)	50.43 ± 10.59	55.97 ± 8.49	0.012
Height (cm)	163.44 ± 8.0	162.42 ± 8.5	0.562
Weight (kg)	64.28 ± 10.41	63.42 ± 9.0	0.693
BMI	23.96 ± 2.65	23.99 ± 2.44	0.971
DM	3 (4.3%)	0	0.550
CHD	5 (7.2%)	5 (16.1%)	0.277
RF	2 (2.9%)	0	1.000
Hypertension	16 (23.2%)	11 (35.5%)	0.200
Stroke	0	1 (3.2%)	0.310
Hyperlipidemia	2 (2.9%)	3 (9.7%)	0.171
**Preoperative medication**			
ß- receptor blocker	11 (15.9%)	9 (29%)	0.130
CCB	12 (17.4%)	3 (9.7%)	0.381
Statin	4 (5.8%)	3 (9.7%)	0.674
Anticoagulant	3 (4.3%)	3 (9.7%)	0.371
Amiodarone	0	0	/
Digoxin	4 (5.8%)	1 (1.6%)	1.000
ACEI, ARB	12 (17.4%)	4 (12.9%)	0.770
Diuretics	38 (55.1%)	20 (64.5%)	0.376
**Preoperative TTE**			
LA (mm)	40.06 ± 7.63	46 ± 11.94	0.015
RA (mm)	35.32 ± 5.54	37.48 ± 11.15	0.312
LVEF (%)	62.57 ± 9.35	59.97 ± 12.45	0.251
CRE (μmol/L)	76.59 ± 15.12	81.87 ± 12.89	0.123
GFR (ml/min/1.73 m^2^)	93.00 ± 13.81	82.02 ± 14.94	0.001
Urea (mmol/L)	5.59 ± 1.32	6.40 ± 1.75	0.012
ALT (IU/L)	21.64 ± 16.16	21.23 ± 9.87	0.896
AST (IU/L)	20.90 ± 9.10	22.00 ± 5.25	0.532
ALP (IU/L)	78.91 ± 34.31	83.42 ± 30.71	0.532
TG (mmol/L)	1.47 ± 1.01	1.55 ± 1.04	0.715
CHO (mmol/L)	4.58 ± 1.04	4.39 ± 0.82	0.352
**Operative details**			
Surgery time (h)	4 (3,4.5)	4 (3,4.5)	0.634
CPB (min)	110 (85,133.5)	110 (96,137)	0.474
Acc (min)	73 (60,103.5)	78 (59,108)	0.864
Mechanical ventilation time (h)	33 (18.5,47)	52 (34,88)	0.001
**Surgery procedures**			
Single valve	34 (49.3%)	7 (22.6%)	0.012
Multiple valve	15 (21.7%)	17 (54.9%)	0.001
CABG	4 (5.8%)	3 (9.7%)	0.674
Aortic replacement	17 (24.6%)	4 (12.9%)	0.183

BMI, body mass index; DM, diabetes mellitus; CHD, coronary heart disease; RF, renal failure; CCB, calcium channel blockers; ACEI, angiotensin converting enzyme inhibitor; ARB, angiotensin-II receptor blocker; TTE, transthoracic echocardiography; LA, left atrium; RA, right atrium; LVEF, left ventricular ejection fraction; CRE, creatinine; GFR, glomerular filtration rate; ALT, glutamate pyruvate transaminase; AST, aspartate aminotransferase; ALP, alkaline phosphatase; TG, triglyceride; CHO cholesterol. CPB, cardiopulmonary bypass; Acc, aortic cross-clamp. CABG, coronary artery bypass graft.

In the preoperative ECG of patients in the two groups, there was a significant difference in the maximum time of the P-wave (167 ± 31 ms vs. 184 ± 37 ms, *P* = 0.018), the SD of the P-wave time (15 ± 7 vs. 19 ± 11, *P* = 0.031), and the PWd (62 ± 28 ms vs. 80 ± 35 ms, *P* = 0.008). However, the minimum time of the P-wave, the average time of the P-wave, the average peak time of the P-wave and the SD of the peak time of the P wave were not different (Table 2).

**TABLE 2:** Comparison of preoperative ECG P-wave characteristic.

Parameters	no-POAF	POAF	*P*-value
Pmax (ms)	167 ± 31	184 ± 37	0.018
Pmin (ms)	105 ± 22	104 ± 19	0.872
Pmean (ms)	133 ± 23	141 ± 25	0.162
Pstd	15 ± 7	19 ± 11	0.031
PWd (ms)	62 ± 28	80 ± 35	0.008
Pptmean (ms)	72 ± 16	73 ± 18	0.923
Pptstd	13 ± 6	14 ± 8	0.432

**FIGURE 4:**
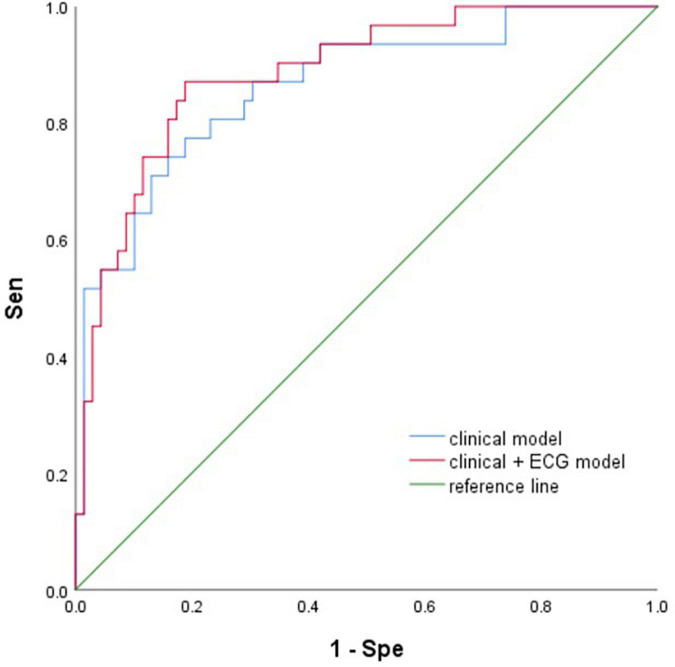
The ROC of two models. Sen, sensitivity; Spe, specificity; ECG, electrocardiogram.

Two multivariate prediction models: (model 1) clinical model with the five selected variables age, sex, LAD, GFR, and mechanical ventilation; and (model 2) ECG and clinical models with eight variables Pmax, Pstd, PWd, age, sex, LAD, GFR, and mechanical ventilation. The AUCs for the two models were 0.86 (95% CI 0.78–0.94) and 0.89 (95% CI 0.82–0.96), respectively (Figure 4).

In scheme A, all patient data were included, and after the hyperparameters of the SVM model were adjusted (penalty Factor C = 0.58, gamma = 3.59), the Ac of the training set was 84%, and the Ac, sensitivity and specificity of the test set were 66, 22, and 74%, respectively. In scheme B, the Ac of the training set was 86%, while the Ac, sensitivity and specificity of the test set were 67, 56, and 78%, respectively, with the penalty Factor C = 95.6 and gamma = 0.83 (Table 3).

**TABLE 3:** The results and parameters of machine learning model.

Scheme	Hyperparameter		Train (0:1)	Test (0:1)	Train set	Test set		
	C	gamma			Ac	Ac	Sen	Spe
A	0.58	3.59	19:19	47:9	0.84	0.66	0.22	0.74
B	95.6	0.83	19:19	9:9	0.86	0.67	0.56	0.78

C, penalty-factor C; Ac, accuracy; Sen, sensitivity; Spe, specificity.

## Discussion

### Differences between wearable long-term electrocardiogram monitoring and conventional monitoring

We found that the detection rate of POAF by wearable long-term ECG monitoring was 31%, while the rate was only 19% through conventional ECG monitoring (Conventional ECG monitoring means 12-lead ECG or continues ECG monitoring after surgery in ICU). Compared with routine clinical monitoring, long-term ECG can significantly improve the detection rate of POAF. Ha et al. (9) found that enhanced rhythm monitoring in patients with a high risk of stroke could improve the detection rate of POAF by continuous ECG monitoring for 30 days after cardiac surgery, which was 10 times higher than that of routine follow-up. Due to the short time of ECG monitoring during postoperative hospitalization, routine methods cannot capture the occurrence of transient POAF.

The 2020 ESC Guidelines suggested that convenient intermittent or systematic monitoring for elderly patients could improve the detection rate of paroxysmal AF. It noted that advances in wearable technology, single-lead electrocardiogram devices and smart devices, could provide a cheap and practical option for AF detection and evaluation (6). The mSToPS (10) results showed that the detection rate of new atrial fibrillation at 4 months through a wearable ECG device was obviously increased, with a reduction in the incidence of clinical end-point events.

At present, the clinical monitoring of POAF is still intermittent and fragmented. The high detection rate and convenience of long-term ECG can greatly capture all kinds of ECG events within 7 days, 30 days or even longer without any obvious burden on patients. Not only can patients with paroxysmal AF and a high risk of POAF be identified preoperatively, but transient POAF can also be timely and accurately screened, which makes it more convenient to adopt targeted prevention and treatment.

### Occurrence mechanism and influencing factors of postoperative atrial fibrillation

Previous studies had proposed a mechanism of POAF, which environmental triggers interact with substrates. When transient postoperative factors act on the sensitive atrial, due to preoperative factors and surgical induction, POAF is triggered (11). Preoperative factors such as age, sex, and underlying diseases may lead to atrial fibrosis and electrophysiological remodeling, thereby increasing sensitivity. Atrial incision and cardiopulmonary bypass can improve the sensitivity of atrial and approach the threshold of POAF. POAF is triggered by the combination of transient autonomic nervous system, immune inflammation response and oxidative stress. Inflammation plays a crucial role in cardiovascular disease (12). Tascanov et al. found that total oxidant status and DNA damage levels were significantly higher in patients with atrial fibrillation. Structural and electrophysiological changes play an important role in the development of AF. Atrial remodeling begins in sinus rhythm long before the onset of AF in patients with recurrent PAF. This is a time-dependent process that can be explained as adaptive changes of myocytes to protect the atrium from external stress. It is therefore important to identify patients with PAF at an early stage (13). Ocak and Tascanov (14). Combined use of troponin I and P-wave dispersion for predicting AF recurrence.

Comparing the baseline data of the two groups, the POAF group was significantly older than the no-POAF group. Most studies (15, 16) reported that the older the patients were, the more obvious their cardiovascular structural and electrophysiological abnormalities were, leading to a non-linear increase in the incidence of POAF. The incidence was significantly higher in patients over 55 years.

LA diameter has been identified as an important risk factor for AF (17, 18). AF is rare when the LA diameter is less than 40 mm, and the risk of AF increases by 39% for each 5 mm increase (18, 19). A gradual increase in LA diameter is associated with the transition from sinus rhythm to paroxysmal AF to permanent AF (19).

Our study found that poor preoperative renal function is associated with the occurrence of POAF. The mechanism of AF induced by renal insufficiency is not completely clear. Myocardial fibrosis may be aggravated by hypertension, volume overload and activation of the renin-angiotensin-aldosterone system (RAAS) (20). In patients undergoing cardiac surgery, the high level of acute systemic inflammation and oxidative stress leads to renal hypoperfusion, which activates the autonomic nervous system and RAAS system and leads to arrhythmias (21, 22).

### Relationship between P-wave and postoperative atrial fibrillation in preoperative sinus electrocardiogram

Even apparently normal hearts have undergone structural and electrical conduction changes as they progressed from sinus rhythm to AF (23). Changes in atrial structure, such as myocardial hypertrophy, fibrosis and enlargement, occur before the occurrence of AF, which all lead to weak electrocardiogram changes in the atria (2). Sinus rhythm cannot completely represent the function of the atria (3). The insignificant changes in the P-wave on ECG reflect local non-sinus atrial electrical activity. These subtle changes can be detected by various algorithms and serve as important factors in predicting the occurrence of AF (7, 24). Many ECG characteristics have been regarded as important predictors, especially P waves.

P wave duration is an important reference for the occurrence of POAF. A shortened atrial refractory period and reduced atrial conduction rate lead to atrial conduction block and a prolonged conduction time, resulting in a prolonged P wave duration (25). PWd has gradually been recognized an important predictor of atrial fibrillation after cardiac surgery and electrical cardioversion (26). The heterogeneity of atrial structure or electrophysiology in the early stage of AF plays an important role in reentry and affects the P wave duration at different times, which is more accurate than using P wave duration alone (27). An increase in PWd suggests that there is heterogeneous electrical activity in different parts of the atria. Chandy et al. (26) analyzed 300 patients who underwent CABG and found that an increase in postoperative PWd compared to preoperative PWd was an independent predictor. Lazzeroni et al. (28) compared the postoperative PWd and P wave duration after CABG and valve surgery, which showed that both had good predictive ability and the PWd variation was higher in the POAF group.

The area of the P wave is also considered to be a marker of atrial structural abnormalities (29).

Besides, under the premise of sinus rhythm, the product of the amplitude depth, and duration of the negative part of the P wave is calculated when the P wave of the V1 lead is bidirectional (30, 31). P wave negative terminal force (PtfV1) is a sensitive marker for judging left atrial enlargement (32). Atrial fibrosis and scarring can indirectly lead to abnormal PtfV1.

Most of the ECGs in previous studies were from public databases or 12-lead ECGs of patients who were retrospectively collected. There are many problems with these approaches, such as whether the routine ECG of several seconds can completely represent the preoperative ECG changes and trends of patients; retrospective collection and database ECG may have omission and bias, which can lead to large errors in ECG feature calculation. Studies on long-term ECG are mostly limited to the screening of events such as AF, and there are few reports on the analysis of its waveform characteristics. Although the long-term data may be affected due to the patient’s wearing mode and various noises, its validity may avoid the problems of short-term conventional ECG, so feature calculation can be carried out more accurately.

In our study, only the P wave characteristics were calculated. Pft is a bidirectional waveform, which requires high signal quality. In actual detection, we found that the long-term ECG data were not very clean, so it was difficult to detect and calculate this feature. The absence of such features may also limit the results. The heart rate variability has become one of the most excellent points to predict the POAF base on 12-lead ECG in the previous studies. In the process of labeling all preoperative ECG data, we found that there was variability in P waveform at different time periods. First of all, it may be because of the inconsistent activity state and autonomic nerve excitability at different times. At night, the sleep state and parasympathetic dominance of the patient lead to negative chronotropic and negative metamorphosis of the heart. During the day, the increased activity of the patient and the sympathetic nerve lead to the increased heart rate, and the increased conductivity of excitement in the heart. Secondly, the patient’s posture change and wearing problems will also lead to the change of waveform, obvious interference or even false performance. In our study, P-wave signs were selected to analyze, however, heart rate variability requires dynamic analysis of patients’ ECG data for 24 h or more, which is more affected by signal quality. Therefore, basing on the advantages and disadvantages of long-term ECG, the current study only calculates the characteristics of part of P waves, and has not introduced dynamic analysis and heart rate variability. In the future research, we will deal with different types of feature group computation and model building.

Our results showed that a model incorporating baseline clinical data and ECG parameters is more effective in predicting POAF than a model using clinical characteristics alone. The model 2 included 5 kinds of clinical information (gender, age, LA, GFR, mechanical ventilation time) and 3 kinds of P wave parameters (Pmax, Pstd, PWd). The AUC was 0.89, and the predictive performance of the model was significantly better than that of a previous study with multiple clinical variables. Preoperative changes in the P wave are important factors for predicting the occurrence of POAF.

With our model, we could identify patients who may be prone to POAF by preoperative ECG. For these patients, it is necessary to strengthen their perioperative management, such as more rhythm monitoring, maintaining the balance of the internal environment, strengthening anti-infection treatment, and appropriately increasing the duration of mechanical ventilation, which can prevent POAF from being triggered by a series of postoperative factors.

### Prediction of postoperative atrial fibrillation by artificial intelligence and machine learning

Artificial intelligence and machine learning can simulate or realize human learning behavior to require new knowledge and skills and reorganizes its existing knowledge structure to continuously improve its performance. In previous studies, ECG data obtained by traditional monitoring methods were analyzed by artificial intelligence and machine learning, but long-term ECG data were used in our study. The larger amount of data and the larger time span make the analysis more specific.

There are many traditional machine learning models, including naïve Bayes, K nearest neighbor, logistic regression, decision making Tree, random forest, XGboosting algorithm, and SVM. Different models have different applications and advantages and disadvantages. SVM was used in our study. SVM is a widely used binary classifier using supervised learning. It is based on the theory of computational learning and structural risk minimization and has obvious advantages in theoretical methods. It has many unique advantages in solving small sample, non-linear and high-dimensional pattern recognition problems and can overcome the curse of dimensionality to a large extent (33, 34). In a binary problem, it mainly introduces the samples that need to be classified into a high-dimensional space and attempts to create an optimal hyperplane to divide the samples into two categories so that the hyperplane can ensure the classification Ac and maximize the area on both sides of the hyperplane. It can ensure that the extremum is the optimal solution, which makes it have good generalization ability.

The penalty factor C and Gamma kernel function are two important hyperparameters in SVM. The parameters set manually before machine learning. The value of the penalty factor C balances the empirical risk (the ability to fit the sample) and the structural risk (the ability to predict the test sample). Although the number of people in the two training sets is the same, the hyperparameter values will change according to the different samples randomly sampled. In scheme A, the model is prone to underfitting if the value of C is small; In scheme B, if C is large, the model is prone to overfitting. Although there is little difference in Ac between the test set and training set of the two groups, the sensitivity in the test set of scheme A is low, which affects the Ac to a certain extent. However, in the more rigorous scheme B, the sensitivity and specificity of the test set are higher, and the test effect is better.

However, the performance of our model is not very good for POAF detection, showing low Ac and a low positive prediction rate. The reason may be as follows: (1) the sample size is small, and the classifier cannot learn the overall distribution of various categories well, so the classification effect is not good. The results of AI and machine learning depend on the quality and quantity of data. Although traditional machine learning can analyze the characteristics of hundreds of ECGs, the results were not as good as those of previous large studies that included exponentially more data points. (2) The types of data were relatively complex. Although all signals were manually calibrated, some parts of the signal were still of poor quality, which affected the results of P wave detection and feature calculation. (3) Current feature analysis was insufficient. Although atrial waves play an important role in the judgment of atrial fibrillation, in fact, waves are susceptible to noise, so additional research on atrial waves in long-term monitoring environments may be beneficial.

Karri et al. (35) compared the predictive performance of 6 machine learning models (random forest, decision tree, logistic regression, K-nearest neighbor, SVM, gradient boosting) and the POAF score for the occurrence of POAF in the ICU after cardiac surgery and found that according to the preoperative clinical data, the performance of machine learning was better than that of the clinical score. The use of AI-equipped medical apps and tools on wearable devices can reduce the dependence on expert care and the cost to the health care system. Perez et al. (36) evaluated the monitoring algorithm of Apple Watch. They found that 34% of patients were confirmed to have atrial fibrillation, and the positive predictive value was 0.84. In addition to smartwatches, photoplethysmography (PPG) measurements of contactless faces and fingertips using a smartphone camera also showed excellent potential for screening and diagnosing atrial fibrillation. Yan et al. (37) used a digital camera and a deep convolutional neural network to prospectively evaluate the feasibility of high-throughput atrial fibrillation detection. Therefore, with the advent of the era of mobile technology, wearable devices with AI would be sensitive to the detection and evaluation of AF, which will be a cheap, non-invasive monitoring method in the future (38).

### Innovation and limitation

The innovation of this study is that there are still few applications of long-term ECG monitoring for POAF. The study explored the use of portable long-term ECG monitoring and machine learning model to predict the occurrence of POAF after cardiopulmonary bypass in patients with preoperative sinus rhythm.

Of course, the limitations of this study are as follows: 1. The included sample size is relatively small, which may affect the results. 2. ECG signal quality limits the effective of machine learning model.

## Conclusion

This prospective, observational study analyzed the differences in long-term ECG parameters in patients undergoing cardiac surgery with preoperative sinus rhythm and evaluated the performance of a machine learning model constructed with P wave characteristics to predict POAF. The results of this study showed that long-term ECG monitoring could significantly improve the detection rate of POAF. The model combining P wave parameters and clinical data performed better in predicting POAF. Machine learning based on P wave parameters can predict the occurrence of POAF.

## Data availability statement

The raw data supporting the conclusions of this article will be made available by the authors, without undue reservation.

## Ethics statement

The studies involving human participants were reviewed and approved by West China hospital. The patients/participants provided their written informed consent to participate in this study. Written informed consent was obtained from the individual(s) for the publication of any potentially identifiable images or data included in this article.

## Author contributions

ZW and CY: methodology. KH, WL, and SL: analysis and writing original draft. LB and CL: data gathering. YX, YL, and HY: review. All authors have read and approved the final version of the manuscript.
